# A Novel Immunocompetent Mouse Model for Testing Antifungal Drugs Against Invasive *Candida albicans* Infection

**DOI:** 10.3390/jof6040197

**Published:** 2020-09-30

**Authors:** Lisa K. Ryan, Amy G Hise, Chowdhury Mobaswar Hossain, William Ruddick, Rezwana Parveen, Katie B. Freeman, Damian G. Weaver, Hema P. Narra, Richard W. Scott, Gill Diamond

**Affiliations:** 1 Division of Infectious Disease and Global Medicine, Department of Medicine, University of Florida College of Medicine, Gainesville, FL 32610, USA; lisa.ryan@medicine.ufl.edu; 2 Department of Pathology, Case Western Reserve University School of Medicine, Cleveland, OH 44106, USA; axh48@case.edu; 3 Medicine Service, Louis Stokes Cleveland VA Medical Center, Cleveland, OH 44106, USA; 4 Department of Oral Biology, University of Florida College of Dentistry, Gainesville, FL 32610, USA; masoomchowdhury@gmail.com (C.M.H.); wruddick@ufl.edu (W.R.); rezparfait@gmail.com (R.P.); 5 Fox Chase Chemical Diversity Center, Inc., Pennsylvania Biotechnology Center, Doylestown, PA 18902, USA; kfreeman@fc-cdci.com (K.B.F.); dweaver@fc-cdci.com (D.G.W.); rscott@fc-cdci.com (R.W.S.); 6 Department of Pathology, University of Texas Medical Branch, Galveston, TX 77555, USA; hpnarra@utmb.edu; 7 Department of Oral Immunology and Infectious Diseases, University of Louisville School of Dentistry, Louisville, KY 40902, USA

**Keywords:** mouse model, in vivo imaging, IVIS, host defense peptide, peptidomimetics, candidiasis, defensing, IL-1R

## Abstract

Disseminated infection by *Candida* species represents a common, often life-threatening condition. Increased resistance to current antifungal drugs has led to an urgent need to develop new antifungal drugs to treat this pathogen. However, in vivo screening of candidate antifungal compounds requires large numbers of animals and using immunosuppressive agents to allow for fungal dissemination. To increase the efficiency of screening, to use fewer mice, and to remove the need for immunosuppressive agents, which may interfere with the drug candidates, we tested the potential for a novel approach using in vivo imaging of a fluorescent strain of *Candida albicans*, in a mouse strain deficient in the host defense peptide, murine β-defensin 1 (mBD-1). We developed a strain of *C. albicans* that expresses red fluorescent protein (RFP), which exhibits similar infectivity to the non-fluorescent parent strain. When this strain was injected into immunocompetent mBD-1-deficient mice, we observed a non-lethal disseminated infection. Further, we could quantify its dissemination in real time, and observe the activity of an antifungal peptide mimetic drug by in vivo imaging. This novel method will allow for the rapid in vivo screening of antifungal drugs, using fewer mice, and increase the efficiency of testing new antifungal agents.

## 1. Introduction

Infection by *Candida* species is the fourth most common nosocomial blood infection in the US (for review, see [1]). The infections are often life-threatening, and mortality remains high despite treatment with multiple anti-fungal agents, ranging from 15–40% for adults [2] and 19–31% for neonates and children [3,4]. Invasive candidiasis (IC) is frequent among very low birth weight babies (up to 20% in cases of extremely low birth weight), with a high mortality rate [5]. Infections are predominantly due to the dimorphic fungus, *Candida albicans*. However, an increasing number of infections are due to other, non-albicans species, including *C. glabrata*, *C. parapsilosis*, *C. tropicalis*, and *C. krusei* as well as the emerging multi-drug resistant strain *C. auris*. This shift has been at least partially attributed to an increasing use of prophylactic antifungal therapy [6]. In addition, there is an increase in resistance to conventional antifungal therapies [7]. Even with the newest class of antifungals, the echinocandins, breakthrough IC is increasingly observed [8]. Thus, the development of new therapeutic agents that exhibit potent activity against all *Candida* species, with a mechanism of action that does not lead to the rapid development of resistance is essential.

During the course of our research developing novel small molecules (whose structures are based on host defense peptides (HDPs)) for the treatment of oral and invasive candidiasis [9,10], we observed that several compounds were potently active against clinical *C. albicans* isolates in the presence of 50% human serum, indicating potential use as systemic antifungal agents. Our study using an invasive candidiasis mouse infection model with a single IV administration of one of the serum-active compounds demonstrated robust, dose-dependent fungicidal activity, superior to active comparator agents [9]. This important proof-of-concept data substantiates an approach to develop novel therapies against systemic fungal infections, an indication of utmost clinical need where substantial mortality is encountered with available anti-fungal medications. We further demonstrated the ability to screen numerous similar compounds using a standard invasive candidiasis model in mice, which allowed for identification of optimal compounds [11].

While numerous in vivo models for invasive candidiasis exist (reviewed in [12]), a standard model used to screen antifungal drugs to target invasive candidiasis involves the immunosuppression of mice with an agent such as cyclophosphamide, which induces neutropenia. This is followed by intravenous injection of the fungal pathogen, to allow rapid dissemination [13]. The candidate drugs are usually injected anywhere from 2 to 24 h after infection. There are usually two different methods for quantification of antifungal drug efficacy: mice are either euthanized at different time points after injection (24–72 h) and total fungal kidney burden is quantified by homogenization and plating on a fungal growth medium, or a survival study is carried out, whereby a larger inoculum is delivered, and mice are observed for mortality over a period of time. A standard antifungal treatment such as fluconazole is included as a positive control for comparison. These methods require large numbers of animals to achieve statistical significance. Furthermore, in order to demonstrate the efficacy over time, groups of mice must be euthanized on consecutive days, to quantify the fungicidal effect. Additionally, there are ethical issues associated with survival studies. In order to assure that mice are not suffering, they must be constantly monitored, and euthanized if signs of morbidity are observed.

In vivo imaging systems (IVIS) have been utilized to follow the course of microorganisms and mammalian cells in animal models and provide the advantage of following the location and multiplication of the invading organism in the same host over time (reviewed in [14]). Their use also decreases the number of animals needed to assess the effect of a treatment on a particular disease. IVIS has been utilized to track *Staphylococcus aureus* [15,16], influenza virus [17] and *Candida albicans* [18]. However, these methods require the injection of luciferin as a substrate for the microbially expressed luciferase. Similarly, this method has been used to visualize *C. albicans* infection in the *Galleria* moth larvae [19]. Other IVIS methods track biofluorescence, such as utilizing red fluorescent protein-expressing viruses [20] or *Aspergillus* [21], and have the advantage of not requiring an exogenous substrate. Here, we report a method using IVIS to follow the response of RFP-labeled *C. albicans* both in *G. mellonella* and in a new mouse model of candidemia, and demonstrate its utility in testing novel antifungal agents.

## 2. Materials and Methods

### 2.1. Materials

The host defense peptide mimetic compound, C6 was synthesized by the Fox Chase Chemical Diversity Center, Inc., Pennsylvania Biotechnology Center, Doylestown, PA, USA as previously described [11]. This compound was dissolved in dimethylsulfoxide (DMSO) (Sigma-Aldrich, St. Louis, MO, USA) at the stock concentration of 10 mg/mL and stored at −20 °C.

### 2.2. Mice

Three strains of mice were used to develop the screening method and to validate the infection model of *Candida albicans*. Female 20-week-old IL-1 receptor (*IL1r1*^−/−)^ mice (The Jackson Laboratory, Bar Harbor, ME), C57Bl/6 wild-type and mouse β-defensin-1 (mBD-1)-deficient (*DefB1*^−/−^) female mice (henceforth referred to as mBD-1^−/−^), developed on a C57Bl/6 background [22] and bred at the University of Florida Animal Care Services facility, were used in the study. All mice were given autoclaved standard rodent diet and sterile water ad libitum, and acclimated for at least 7 days after transfer from The Jackson Laboratories or the transgenic breeding facility at the University of Florida. Some mice were housed in HEPA filter-covered microisolator cages in ventilated racks in the AAALAC accredited facilities at Case Western Reserve University School of Medicine under approved protocols from the Institutional Animal Care and Use Committee.

### 2.3. Yeast Strain

*Candida albicans* strains SC5314 (a lab isolate originally obtained from a clinical disseminated infection) and GDH2346 (originally isolated from a patient with denture stomatitis) and GDH2346-RFP were cultured on YPD (1% yeast extract, 2% peptone, 2% dextrose, pH 5.7) agar (DIFCO Laboratories, Detroit, MI, USA) at 37 °C. Stock cultures were then prepared and stored in glycerol at −80 °C. The frozen stocks were then grown on YPD plates and kept at 4 °C. Prior to each experiment, a stock inoculum suspension of *C. albicans* was prepared in YPD broth and incubated at 30 °C with shaking at 200 rpm for 12 h. The culture was washed twice with sterile 1× phosphate buffered saline (PBS), counted with a hemocytometer, and diluted with PBS to get the desired concentration of 3.5 × 10^5^ cfu/mL. The inoculum concentration was verified by both optical density and by dilution plating onto YPD agar prior to infection. Fluorescence was also verified in vitro prior to infection.

### 2.4. Plasmid Construction and Electroporation of C. albicans Strain GDH2346

The plasmid cIP-ADH1p-mCherry [23] was kindly provided by Neta Dean (SUNY-Stonybrook). This plasmid contains codon optimized mCherry under a constitutive ADH1 promoter and used URA3 marker for selection. Since URA3 selection cannot be used in the GDH2346 wild-type strain, a *Candida* codon optimized hygromycin B resistance cassette driven by the ACT1 promoter was amplified from pYM70 [24] and cloned into the AatII site, generating a plasmid construct, named AH070, and capable of constitutively expressing both mCherry and Hygromycin B. Five micrograms of plasmid AH070 was electroporated into *C. albicans* strain GDH2346 following the protocol described earlier [25]. Briefly, GDH2346 was grown in YPD medium at 30 °C to an OD_600_ of 1.6–2.2. The cells were made electro-competent using 1 M lithium acetate pH 7.5 and resuspended in 1M sorbitol. Electroporation was performed in Bio-Rad electroporator in 0.2 cm cuvette at 1.8 kV. Immediately after electroporation, the cells were recovered by growing them in 1 mL of YPD medium at 30 °C for 4 h. Then, 100 µL aliquots of recovered cells were plated on YPD agar supplemented with 300 ug/mL hygromycin B (Sigma-Aldrich) and were grown at 30 °C for 1–2 days. The resistant colonies were picked and screened for RFP and hygromycin. Clonal population of positive clones were made by streaking colonies twice continuously on fresh YPD agar plus hygromycin (300 ug/mL) plates. The expression of RFP was confirmed at both yeast and hyphal stages by microscopy.

### 2.5. Infection

#### 2.5.1. Murine Disseminated Candidiasis and Treatment

*C. albicans* infection was performed by injecting C57Bl/6 or mBD1^−/−^ mice (*n* = 5/group) with 0.1 mL of 3.5 × 10^5^ cfu/mL in PBS intravenously (i.v.) in the dorsal side of the tail. C6 was dissolved in 20% kleptose at 2 mg/mL concentration and sonicated for 10 min. The compound was injected subcutaneously (subQ) in the dorsal side of the neck 2 h after infection. The mice were humanely killed by CO_2_ asphyxiation and both kidneys were collected from five mice of infected control group 2 h after infection and from the remaining control and drug-treated mice in the study at 14 days after infection. Two kidneys were weighed and combined in a sterile homogenizer tube/mouse. Ten milliliters of sterile PBS was added to each tube and the contents uniformly homogenized with a tissue homogenizer (Ultra-TURRAX Tube Drive, IKA Works, Inc., Wilmington, NC, USA). Serial dilutions of the tissue homogenates were conducted, 0.1 mL aliquots were spread on YPD agar plates, and the plates incubated at 35 °C overnight. cfu/g kidneys were determined from colony counts.

#### 2.5.2. Murine Oropharyngeal Candidiasis (OPC)

Mouse OPC studies were done following published protocols [26,27,28]. Briefly, after 5 days of tetracycline treatment (in drinking water) C57BL/6 (*n* = 6/group) or *IL1r1*^−/−^ (*n* = 12/group) mice were infected orally with 5 × 10^6^ cfu of GDH2346 or GDH2346-RFP (*n* = 3/mouse strain). After 3 days, the mice were humanely euthanized by CO_2_ asphyxiation, the oral cavity inspected and the degree of infection was rated using a previously published scoring system [26]. Organs were harvested, weighted, homogenized, serially diluted in sterile PBS and plated on Sabouraud dextrose (SD) agar and cfu counted after 48 h incubation at 37 °C. To validate the ability to visualize the GDH2346-RFP strain histologically, *IL1r1*^−/−^ mice were infected orally with 5 × 10^6^ cfu GDH2346-RFP for 3 days and the tongues were harvested and 5-µm sections cut on a cryotome, stained for DNA (DAPI) and imaged using a fluorescent microscope (Leica, Buffalo Grove, IL, USA).

#### 2.5.3. Galleria mellonella Infection

*C. albicans* GDH2346-RFP, was grown in liquid culture to an OD_600_ of 0.2, and washed twice with PBS, and diluted to a concentration of 2.5 × 10^5^ cfu/mL. *G. mellonella* larvae (300–400 mg) were immobilized by cooling to 4 °C, and 10 µL *Candida* suspension was injected under the last left proleg. Injected larvae were incubated at 37 °C in the dark for 24 h prior to imaging. No lethality was observed at this concentration.

### 2.6. In Vivo Imaging Quantification

Mice were anaesthetized by isofluorane and visualized in a Xenogen IVIS (Perkin Elmer-Caliper Life Sciences). Mice were imaged daily for 14 days. Prior to imaging, hair was removed from the ventral side of the mice using a commercial depilating cream (CVS Pharmacy, Newberry, FL, USA). Mice were anesthetized with isoflurane in the IVIS imager and imaged for fluorescence with the following settings: Exposure time, 1 s. Medium binning, F-stop = 2, excitation: 570 nm, emission: 620 nm.

Standard photographic images were directly overlaid on matching fluorescent images. Fluorescence was quantified with the Living Image software version 4.1, using the region of interest (ROI) tool, where fluorescence from the bare region was measured. Background fluorescence was assessed in an uninfected mBD-1^−/−^ mouse and subtracted from each test mouse image. IVIS fluorescent images were quantified by the number of photons per second that leave a square centimeter of tissue and radiate into a solid angle of one steradian (sr). Data were collected and quantified as mean radiance, which are expressed as mean ± standard deviation p/s/cm^2^/sr.

*G. mellonella* larvae were similarly imaged, having been immobilized by cooling for 5 min on a freezer block.

### 2.7. Statistical Analysis

Comparison of the means was performed using unpaired Student’s *t*-tests or Mann–Whitney test (for unequal groups) using commercial software (GraphPad Prism version 8.0) with *p* values less than 0.05 considered statistically significant.

### 2.8. Ethical Statement

All animals were maintained in accordance with American Association for Accreditation of Laboratory Care criteria. The animal experiments were designed and conducted upon approval of the Institutional Animal Care and Use Committees (IACUC) (protocol number 201408371, 29 May 2014), University of Florida, USA and Case Western Reserve University (protocol number 2014–0023, A.G.H.) strictly in accordance with the guidelines of IACUC.

## 3. Results

### 3.1. RFP-Candida Validation

**Figure 1:**
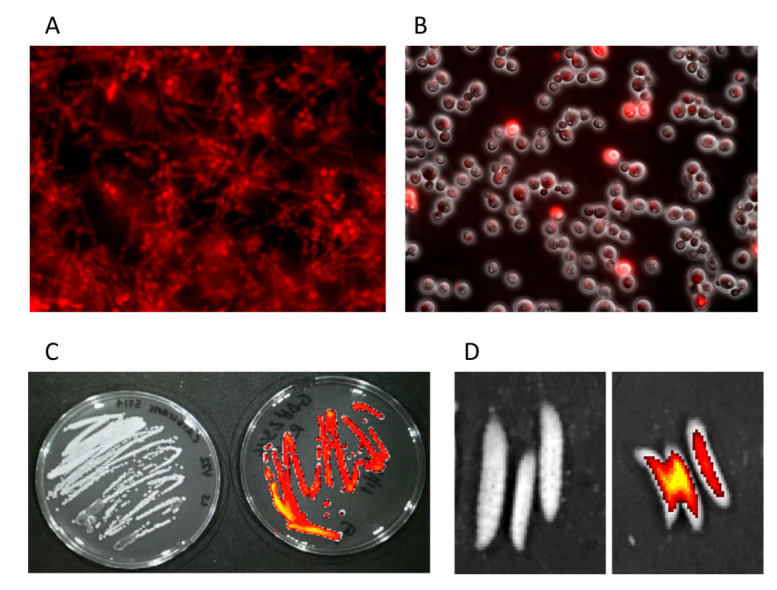
Visual imaging of *C. albicans* GDH2346. (**A**) GDH2346-RFP, grown for 24 h in hyphal permissive media, wet slide image on fluorescent microscope (40× magnification). (**B**) GDH2346-RFP yeast cells imaged with light phases and fluorescent microscopy, showing RFP expression (63×). (**C**) 24-h cultures of *C. albicans* SC5314 (left) and GDH2346-RFP (right), grown on YPD agar plates, and visualized in the IVIS. (**D**) *G. mellonella*, injected with PBS (left) or *C. albicans* GDH2346-RFP (right) after 24 h, visualized in the IVIS.

To validate RFP expression in yeast cells, GDH2346-RFP was grown for 16 h in YPD broth, pelleted and washed in PBS and a wet slide prepared for fluorescent microscopy. As shown in Figure 1, fluorescence is observed in all stages of *Candida* growth. We observe fluorescence in this strain microscopically, both in hyphal permissive medium (Figure 1A), and in the yeast form (Figure 1B). To assess RFP expression in a biofilm, plates were prepared of SC5314 or GDH2346-RFP growing on SD agar. Figure 1C shows the in vitro red fluorescence of the RFP-producing *C. albicans* GDH2346-RFP compared with the lack of red fluorescence of *C. albicans* SC5314. To demonstrate the ability to visualize the *Candida* strain during an in vivo infection, we injected 2.5 × 10^3^ cfu of GDH2346-RFP into *G. mellonella* larvae, and imaged the larvae after 24 h. Figure 1D shows strong fluorescence in the *Candida*-injected larvae compared with control.

**Figure 2:**
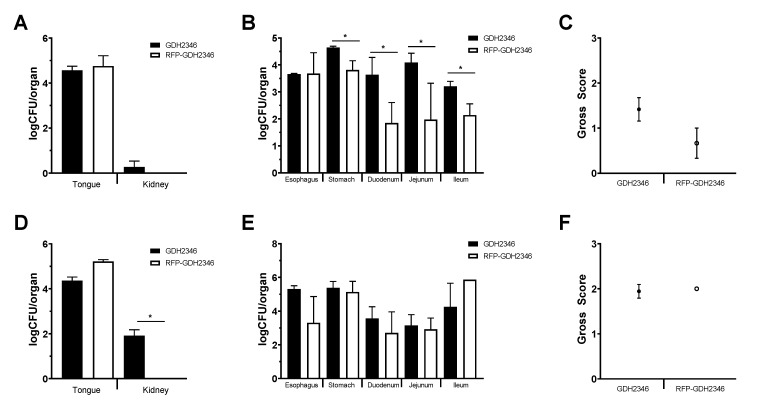
OPC infection of mice infected with GDH2346 versus GDH2346-RFP. Quantitative fungal burdens of tongues and kidneys of mice infected for 3 days (**A**,**D**); quantitative fungal burdens of esophagus and stomach, duodenum, jejunum and ileum (**B**,**E**); gross clinical pathology scores (**C**,**F**). Wild-type mice (*n* = 6 for GDH236; *n* = 3 for RFP) are shown in the upper panels (**A**–**C**) and *IL1r1*^−/−^ mice (*n* = 12 for GDH236; *n* = 3 for RFP) are shown in the lower panels (**D**–**F**). *, *p* < 0.05.

The infectivity of *C. albicans* GDH2346-RFP in mice was then evaluated using the non-immunosuppressed oral candidiasis mouse animal model described by Tomalka et al. [27]. C57BL/6 or IL-1R1 deficient mice were infected orally with 5 × 10^6^ cfu of GDH2346 or GDH2346-RFP as described. After 3 days, the mice were euthanized and the oral cavity was assigned a clinical score to indicate the degree and severity of visual infection of the tongue, buccal surfaces and palate [26]. Organs including the tongues, kidneys, esophagus, stomach, duodenum, jejunum and ileum were harvested, weighed, homogenized and cfu measured by serial plating on SD agar. As shown in Figure 2, the *C. albicans* GDH2346-RFP strain shows comparable fungal burdens in the tongue (B6: *p* = 0.679; *IL1r1*^−/−^ *p* = 0.088), kidney (B6: *p* = 0.517; *IL1r1*^−/−^ *p* = 0.022) (Figure 2A) compared with GDH2346. Although the fungal burdens in the tongues of wild-type compared to *IL1r1*^−/−^ mice were not statistically different (GDH: *p* = 0.0431; GDH-RFP: *p* = 0.428), as expected there was higher fungal burdens in the kidneys of *IL1r1*^−/−^ compared to wild-type mice infected with GDH2346 (*p* = 0.0013). Both strains of mice had zero colonies in the kidneys when infected with GDH2346-RFP. There was less colonization of the stomach (*p* = 0.036), duodenum (*p* = 0.036), jejunum (*p* = 0.036) and ileum (*p* = 0.036) in the wild-type mice infected with the GDH2346-RFP compared to the GDH2346 (Figure 2C) but these organs were comparable in the *IL1r1*^−/−^ mice (*p* = 0.800, 0.700, 0.800 and 0.423 respectively). In both wild-type and *IL1r1*^−/−^ mice, the esophagus had comparable fungal burdens with both strains of *Candida* (B6 *p* = 0.536; *IL1r1*^−/−^ *p* = 0.700). When comparing the fungal burdens of organs of wild-type B6 mice compared to *IL1r1*^−/−^ mice; there is generally a trend of higher fungal burdens in the *IL1r1*^−/−^ mice (esophagus GDH: *p* < 0.0001, esophagus GDH-RFP: *p* = 0.840; stomach GDH: *p* = 0.0431, stomach GDH-RFP: 0.132; duodenum GDH: *p* = 0.886, duodenum GDH-RFP: *p* = 0.389; ileum GDH: *p* = 0.031, ileum GDH-RFP: *p* = 0.449; jejunum GDH: *p* = 0.129, jejunum GDH-RFP: *p* = 0.016). The clinical severity score was also similar in wild-type (*p* = 0.290) and *IL1r1*^−/−^ mice (*p* = 0.874) infected with the two fungal strains, although the clinical score was significantly higher in the *IL1r1*^−/−^ mice compared to wild-type mice infected with GDH2346-RFP (*p* = 0.016) but not GDH2346 (*p* = 0.070).

**Figure 3:**
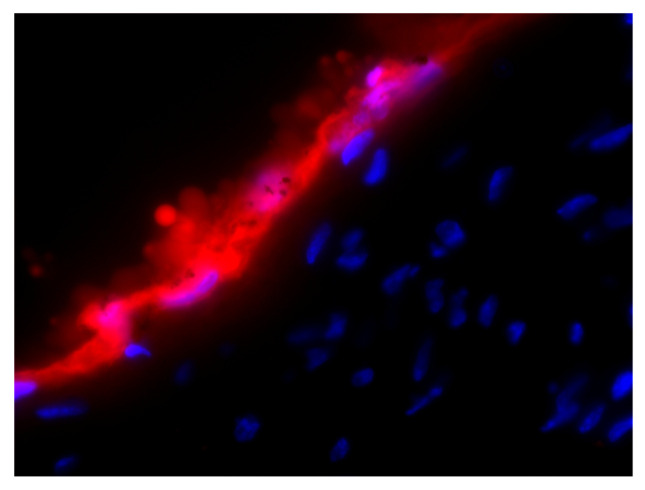
In vivo visualization of RFP expressing *Candida*. Histology of a GDH2346-RFP OPC infected IL1r1^−/−^ mouse showing RFP expression in the fungal biofilm at the dorsal surface of the tongue (5 µm section stained with DAPI and imaged with a scanning fluorescent microscope (63×)).

To assess the ability of the RFP expressing strain to be visualized in vivo, IL-1R1 deficient mice were infected orally using the OPC model. This strain was chosen to maximize the mucosal biofilm as we have previously shown that mice lacking IL-1R1 are significantly more susceptible to mucosal infection [26]. As shown in Figure 3, the dorsum of the tongue shows RFP staining at the location in the stratum corneum of the *Candida* biofilm. DAPI-stained nuclei of the multilayered epithelium can be visualized in blue.

### 3.2. mBD-1^−/−^ Candida albicans Mouse Model

**Figure 4:**
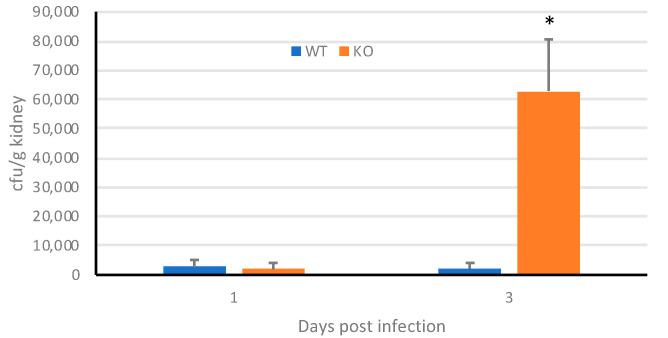
Growth of *C. albicans* in mBD-1^−/−^ mice. Female mice (C57BL/6 and mBD-1^−/−^, *n* = 5 per group) were injected with 3.5 × 10^5^ cfu *C. albicans* and euthanized on days 1 and 3 post infection. Kidneys were homogenized and viable *Candida* were quantified by dilution plating. WT, C57Bl/6; KO, mBD1^−/−^. Data are shown as mean +/– SEM. *, *p* = 0.009.

Mice deficient in β-defensin 1 are susceptible to invasive infections with *C. albicans* after infecting the oral mucosa [27]. Therefore, we examined the potential use of the mBD-1^−/−^ mouse for in vivo imaging of GDH2346-RFP using standard tail vein injection. We injected 3.5 × 10^5^ cfu into immunosufficient female wild-type and mBD-1^−/−^ mice, which resulted in a non-lethal, systemic infection. By day 3 we observe a significant infection in the mBD-1^−/−^ mice compared to the wild-type mice (Figure 4). A similar systemic infection was observed in male mBD-1^−/−^ mice by day 3 (Supplemental Figure S1). Weight loss following infection with *C. albicans* GDH2346 did not exceed 7% (Supplemental Figure S2), thus this infection system represents a useful model for systemic candidiasis.

**Figure 5:**
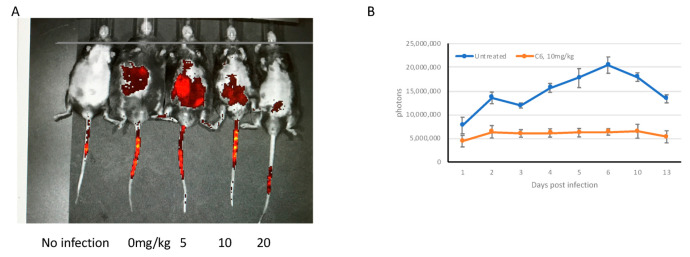
Fluorescence of mice infected with *C. albicans* GDH2346-RFP and treated with C6. (**A**). Male mBD-1^−/−^ mice were injected with 3.5 × 10^5^ cfu *C. albicans* GDH2346-RFP, followed by either PBS or C6 after 2 h. Mice were visualized by IVIS over the course at day 10, and fluorescence was visualized as described. (**B**). Male mBD-1^−/−^ mice (*n* = 5 per group) were injected with 3.5 × 10^5^ cfu *C. albicans* GDH2346-RFP, followed by either PBS or 10 mg/kg C6 after 2 h. Mice were visualized and fluorescence was quantified daily over the course of 13 days.

To use this model to visualize the effects of antifungal drugs, male mBD-1^−/−^ mice were infected with *C. albicans* GDH2346-RFP, followed by injection with either PBS or increasing concentrations of the HDP mimetic compound C6 two hours later. This compound has been shown to exhibit potent antifungal activity in a mouse model of disseminated candidiasis [11]. Mice were imaged after 10 days. The results showed a dose-dependent reduction in fluorescence in the C6-treated mice to background (Figure 5A). While we could not calculate a statistically significant correlation between fluorescence and kidney burden, viable colonies were observed in homogenized kidneys from the untreated mice, while no colonies were seen in the mice treated with 20 mg/kg C6. To demonstrate the ability to quantify the fluorescence over time, mice were infected with GDH2346-RFP, followed by injection with either PBS or 10 mg/kg C6, and fluorescence was quantified daily for 13 days. The results in Figure 5B show that efficacy of the drug can be observed at each time point.

## 4. Discussion

We have demonstrated earlier that small molecule mimics of host defense peptides exhibit potent antifungal activity, both in vitro and in vivo. Major advantages of these molecules over the parent peptides upon which they are designed include the fact that they are inexpensive to synthesize and modify, and that they are protease-resistant, increasing their bioavailability. In addition, similar to the peptides, they are membrane-active, which makes it more difficult for microbes to develop resistance. We have thus been able to screen numerous mimetics both in vitro and in vivo to identify lead compounds for further development as antifungal agents against both oral and disseminated candidiasis [9,10,11,29]. However, testing numerous compounds in vivo requires large numbers of mice, with multiple time points to quantify relative activity compared with standard antifungal agents as controls.

One early screening method involves injection of *Candida* into the larvae of the *Galleria* moth [30]. However, even in the cases where drugs are tested this way, it is primarily a qualitative assay, where the output is survival. While bioluminescent *C. albicans* were visualized in *G. mellonella*, after injection with the substrate Coelenterazine [19], our results here demonstrate that it is possible to obtain quantitative data in this invertebrate model using an RFP-expressing strain of *Candida*, without the need for a second injection of a substrate.

While most mouse models of localized and disseminated *Candida* infections require immunosuppression, the use of immunosuppressive agents could potentially interfere in the interpretation of data, especially when testing novel antifungal drugs. This is the first report utilizing the mBD-1 deletion mutation to model systemic fungal infection and to test antifungal therapy. Murine β-defensin 1 has already been shown to participate in the innate defense against numerous microbial infections, where its deficiency makes mice more susceptible to bacterial [22,31], viral [32,33] and oral Candidal infections [27]. Here, we show that its deficiency clearly allows rapid dissemination of *Candida* in the absence of exogenous immunosuppressive agents, without lethality, using a relatively low inoculum. The use of this mBD-1^−/−^ model could thus allow more accurate pre-clinical screening of novel antifungal drugs.

Previously, the importance of IL-1 signaling in antifungal defense has been demonstrated in multiple murine models of *Candida* infection including intravenous and mucosal models. [26,34,35,36]. In the current study we observed more dissemination with the parent GDH2346 strain than with the GDH2346-RFP strain as measured by fungal burdens in the kidneys in *IL1r1*^−/−^ mice. Previously we have shown that the IL-1R1 deficient mice are more susceptible to systemic dissemination in our OPC model using the GDH-2346 strain [26] including higher oral fungal burdens, although differences were more notable at 7 days of infection rather than the 3 days shown here. Further testing of the GDH2346-RFP strain in the IL-1R1 deficient mice will be needed to confirm whether this is a characteristic of the fungal strain, related to the time course of the experiment or other factors. As we and others have shown, the *IL1r1*^−/−^ mouse model provides an opportunity for consistent oral as well as GI colonization with *Candida albicans* which may be useful for validation of anti-fungal drugs in a mucosal model. We validated the ability to visualize the mucosal biofilm using immunofluorescent microscopy and show comparable mucosal fungal burdens between the parent and RFP-labeled fungal strains in the IL-1R1 deficient mice. Although we did not specifically evaluate the *IL1r1*^−/−^ mucosal model in anti-fungal drug testing, the utility of this model would be predicted to be valuable for this purpose, allowing for visualization of mucosal biofilms and traditional methods of quantifying fungal burdens and generally higher fungal burdens than wild-type mice. While the mBD-1^−/−^ model infected with the *C. albicans* GDH2346-RFP strain itself is equally effective in observing oral mucosal and systemic mucosal infections, the disadvantage of the mucosal models relative to the intravenous infection models is the absence of the ability for high throughput screening and real time quantification of infection using IVIS.

Beta defensin-1 is important in the innate host defense against infection and the effect of its deletion seems to differ depending on the type of infection. Interestingly, the difference in *Candida* burden between wild-type and mBD-1^−/−^ mice was not observed until 3 days after infection. This is in contrast to the effect of mBD-1 deficiency on the susceptibility to *Bordetella bronchiseptica* bacterial infection, where a difference was only observed at 24 h [31]. Deletion of mBD-1 in mice also rendered them more susceptible to mouse-adapted Hong Kong/1968/H3N2 influenza A infection; mBD-1^−/−^ mice had greater leukocyte infiltration into the lungs, body weight loss and mortality compared with wild-type C57Bl/6 mice [37]. Significant differences in body weight loss, but not virus titers, were observed beginning on day 2 and continued throughout the infection. Lung pathology and mortality also differed 5 days after infection with influenza [37].

The differences in the pathology and the numbers of microorganisms in these three types of infection models could reflect differing roles of β-defensin-1 in the innate defense against viral, fungal and bacterial infections. In the infection model for *B. bronchiseptica*, mBD-1 coordinates with mBD-3 to provide innate host defense against infections in the trachea (31). However, in the viral and fungal models, deletion of mBD-1 alone had significant effects on infection. This may reflect the location and degree of gene expression of beta-defensin-1 in certain cell types, which differ in the pathological response to each type of infection. Human BD-1, for example, is differentially expressed throughout the epithelial cells in the mucosal system and in monocytes, neutrophils and plasmacytoid dendritic cells, and this differential expression and modulation by infectious organisms could likely be affecting the immunopathology of these infections [37].

The mBD-1^−/−^ mouse/*Candida albicans* GDH2346-RFP infection model allowed us to test the ability to quantify infection in real time using fluorescently expressing *Candida*. Previously, Vande Velde et al. [18] utilized a bioluminescent strain of *C. albicans*, and was able to visualize the infection using the IVIS. However, this method required both immunosuppression, and the injection of the substrate for the luciferase enzyme. The method described here allows a real-time determination of infection in a non-immunosuppressed mouse model of disseminated candidiaisis. Further research will be necessary to determine the optimal method to directly correlate quantifiable fluorescence with viable cfu of *Candida* in the kidney.

Thus, the development of this novel strain of *C. albicans* that constitutively expresses RFP, provides a useful tool to both reduce the number of mice necessary for these studies, as well as allowing for rapid screening of new drugs. Our results demonstrate that this fluorescent strain can be used in conjunction with the mBD1^−/−^ strain of mice to provide a useful method for testing the efficacy of all classes of antifungal agents in disseminated infections as well as with *IL1r1*^−/−^ mice in mucosal models of infection.

## 5. Conclusions

The method presented here represents a new advance, by not requiring the injection of either luciferase substrate or an immunosuppressive drug. In addition, the use of RFP provides a broader emission spectrum, which can allow for more accurate quantification, and thus can increase the efficiency of testing new antifungal agents to treat *Candida* infections.

## Supplementary Materials

The following are available online at https://www.mdpi.com/2309-608X/6/4/197/s1, Figure S1: Growth of C. albicans in male mBD1^−/−^ mice, Figure S2: Weight loss in *C. albicans* infection model.
