# Supplementary material for: A Novel Immunocompetent Mouse Model for Testing Antifungal Drugs Against Invasive Candida albicans Infection
Source: J Fungi (Basel). 2020 Sep 30;6(4):197. doi: 10.3390/jof6040197 (PMC7712810; doi:10.3390/jof6040197)
Supplement: Supplementary file 1 [file jof-06-00197-s001.pdf]

Supplemental figures

Supplemental figure 1

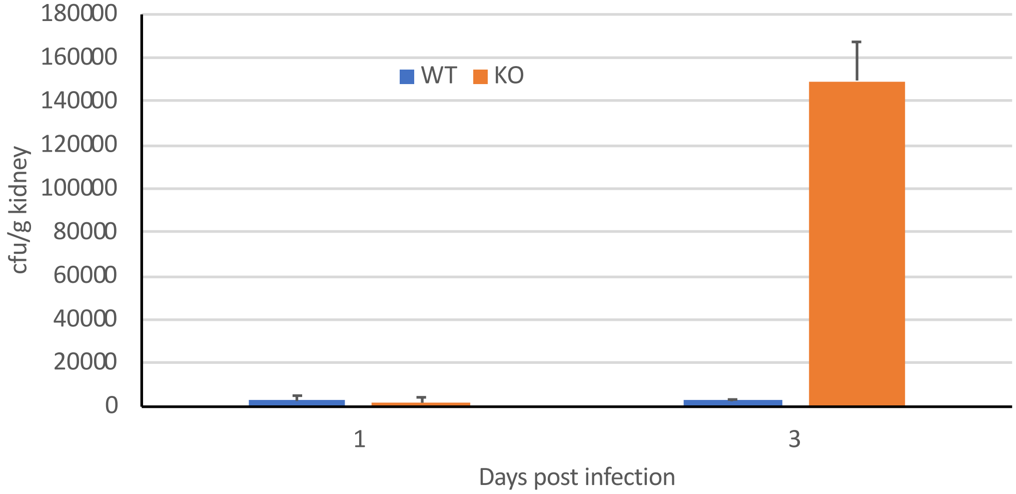

Supplemental Figure 1. **Growth of *C. albicans* in male mBD1$^{-/-}$ mice.** Mice (female C57BL/6 and male mBD-1$^{-/-}$, n=5 per group) were injected with 3.5 x 10^5 cfu *C. albicans* and euthanized on days 1 and 3 post infection. Kidneys were homogenized and viable *Candida* were quantified by dilution plating. WT, C57Bl/6; KO, mBD1$^{-/-}$. Data are shown as mean +/- SEM.

Supplemental figure 2

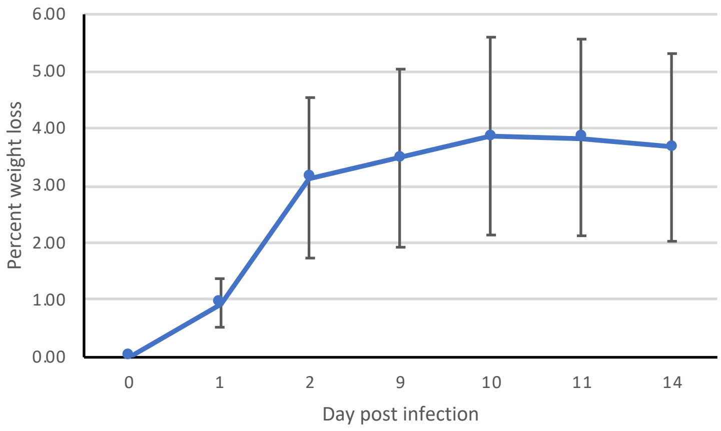

Supplemental Figure 2. **Weight loss in *C. albicans* infection model.** Mice (male mBD-1$^{-/-}$, n=5 per group) were injected with 3.5 x 10^5 cfu *C. albicans* GDH2346-RFP and weighed over the course of the infection. Percent weight loss is calculated from day 0. Data are shown as mean +/- SEM.
